# Impact of Disability on Postoperative Outcomes After Gastrointestinal Cancer Surgery

**DOI:** 10.1245/s10434-025-16904-x

**Published:** 2025-01-16

**Authors:** Shahzaib Zindani, Mujtaba Khalil, Selamawit Woldesenbet, Zayed Rashid, Abdullah Altaf, Jun Kawashima, Austin Schenk, Timothy M. Pawlik

**Affiliations:** Department of Surgery, The Ohio State University Wexner Medical Center and James Comprehensive Cancer Center, Columbus, OH USA

**Keywords:** Disability, Cancer, Surgical outcomes, Healthy days at home

## Abstract

**Introduction:**

Approximately 61 million individuals in the United States have a disability and face unique challenges, resulting in healthcare disparities.

**Objective:**

We aimed to evaluate the impact of disability on postoperative outcomes and number of healthy days at home (HDAH).

**Methods:**

Patients who underwent surgery for gastrointestinal (GI) cancer between 2017 and 2020 were identified using the Medicare database. Multivariable regression models were used to examine the association between disability and postoperative complications, discharge disposition, and the number of HDAH.

**Results:**

A total of 72,452 individuals underwent GI cancer surgery (pancreas: *n* = 7614, 10.5%; hepatobiliary: *n* = 4994, 6.9%; colorectal: *n* = 59,844, 82.6%). Median patient age was 75 years (interquartile range 71–81) with most patients being female (*n* = 37,167, 51.3%). Overall, 5432 individuals (7.2%) had a disability. Following surgery, patients with a disability were more likely to experience complications (4.6% vs. 3.3%), be discharged to a skilled nursing facility (SNF; 26.6% vs. 12.3%), and experience hospital readmission (20.0% vs. 13.5%) [all *p* < 0.001]. Consequently, individuals with disabilities were more likely to spend fewer (<20th percentile) HDAH (33% vs. 19.2%) [all *p* < 0.001]. On multivariable analysis, disability was associated with higher odds of complications (odds ratio [OR] 1.36, 95% confidence interval [CI] 1.19–1.56) and hospital readmission (OR 1.55, 95% CI 1.44–1.66). Additionally, disability was associated with higher odds of spending fewer HDAH (OR 1.88, 95% CI 1.77–1.99).

**Conclusion:**

Following GI cancer surgery, individuals with disabilities had a higher risk of complications and spent fewer HDAH. There is a need for targeted interventions to improve the care of patients with disabilities and ensure equitable oncological and surgical outcomes.

**Supplementary Information:**

The online version contains supplementary material available at 10.1245/s10434-025-16904-x.

While there have been remarkable advancements in cancer care, including new treatments, early detection methods, and personalized therapies, disparities in access and outcomes persist. Individuals from marginalized communities often face substantial barriers, such as a lack of insurance, limited availability of specialized facilities, and reduced access to preventive screenings.^1,2^ Socioeconomic factors and geographical location can further intensify these issues, leading to greater healthcare inequality. These barriers can result in worse health outcomes and lower long-term survival among marginalized populations of patients.^3^ As such, there is an urgent need to implement strategies that ensure equitable access to the latest cancer treatments as a means to mitigate disparities in surgical oncology outcomes.

Individuals with disabilities represent one of the largest underserved groups in the United States (US). According to the US Centers for Disease Control and Prevention (CDC), 61 million Americans currently have at least one disability, and this number is expected to steadily increase in the coming years.^4,5^ Of note, individuals with disabilities face several barriers to equitable healthcare within a system that is primarily designed for able-bodied individuals.^6,7^ Patients may encounter physical obstacles when trying to access clinics and hospitals, communication challenges due to hearing or visual impairments, and financial difficulties stemming from reduced employment and educational opportunities.^8–10^ Previous reports have demonstrated that individuals with disabilities are less likely to engage in screening and preventive services and are less likely to have had a routine doctor visit in the past year.^11,12^ For instance, Khan et al. noted that female patients with disabilities were 23% less likely to undergo breast cancer screening.^11^ Similarly, Rafaqat et al. reported that individuals with disabilities were less likely to undergo minimally invasive surgery.^13^

Previous studies have demonstrated that disability impacts outcomes for patients hospitalized due to chronic medical conditions or emergency surgeries.^14^ However, these findings may not apply to cancer care, which is inherently complex and requires adherence to intricate treatment plans. Therefore, we sought to investigate the impact of disability on postoperative outcomes and the number of healthy days at home (HDAH) among patients undergoing a surgical procedure for a gastrointestinal (GI) cancer.

## Methods

### Data Source, Study Population, and Cohort Selection

The Medicare Standard Analytic Files (SAFs) were queried using International Classification of Diseases, Tenth Edition (ICD-10) codes to obtain data on patients who underwent an operative procedure surgery for a GI cancer indication. The study included individuals aged 65 years and older who underwent hepatectomy, pancreatectomy, colectomy, or proctectomy between 2017 and 2020. Patients who were not enrolled in Medicare Part A or B during the month of the surgery or who received payments from a health maintenance organization (HMO) were excluded. If a patient underwent multiple surgical procedures within the study period, only the first procedure was included. Disability was defined according to the CDC Disability and Health Data System and included intellectual impairment, mobility impairment, hearing impairment, and visual impairment. A validated algorithm with high sensitivity and specificity to identify disability in administrative datasets was used to establish a preoperative diagnosis of disability.^13,14^ This algorithm utilized ICD-10 codes to identify at least two diagnoses of disability within an outpatient setting and one diagnosis of disability within an inpatient setting (electronic supplementary material [ESM] Table 1). The Institutional Review Board at Ohio State University approved this study and waived the requirement for informed consent since the data were limited.

**Table 1:** Baseline characteristics of patients

Characteristics	Total	Disability		*p* value
	[*n* = 72,452]	No [*n* = 67,020, 92.5%]	Yes [*n* = 5432, 7.5%]	
Age, years [median (IQR)]	75 (71–81)	75 (71–80)	77 (72–82)	<0.001
Sex				0.99
Male	35,285 (48.7)	32,640 (48.7)	2,645 (48.7)	
Female	37,167 (51.3)	34,380 (51.3)	2,787 (51.3)	
CCI				<0.001
<2	33,137 (45.7)	31,175 (46.5)	1962 (36.1)	
≥2	39,315 (54.3)	35,845 (53.5)	3470 (63.9)	
Ethnicity				<0.001
White	64,152 (88.5)	59,384 (88.6)	4768 (87.8)	
Black	4217 (5.8)	3844 (5.7)	373 (6.9)	
Hispanic	599 (0.8)	539 (0.8)	60 (1.1)	
Other	3484 (4.8)	3253 (4.9)	231 (4.3)	
Region				<0.001
Midwest	17,460 (24.1)	15,940 (23.8)	1520 (28)	
Northeast	13,522 (18.7)	12,458 (18.6)	1064 (19.6)	
South	28,994 (40)	26,955 (40.2)	2039 (37.5)	
West	12,476 (17.2)	11,667 (17.4)	809 (14.9)	
Area				0.28
Metropolitan	55,478 (76.6)	51,351 (76.6)	4127 (76)	
Non-metropolitan	16,971 (23.4)	15,666 (23.4)	1305 (24)	
Teaching status				<0.001
Non-teaching	28,210 (38.9)	26,229 (39.1)	1981 (36.5)	
Teaching	44,242 (61.1)	40,791 (60.9)	3451 (63.5)	
SVI				0.64
Low	24,527 (33.9)	22,679 (33.8)	1848 (34)	
Moderate	24,178 (33.4)	22,396 (33.4)	1782 (32.8)	
High	23,747 (32.8)	21,945 (32.7)	1802 (33.2)	
Cancer type				0.86
Biliary tract	2649 (3.7)	2460 (3.7)	189 (3.5)	
Liver	2345 (3.2)	2177 (3.2)	168 (3.1)	
Pancreatic	7614 (10.5)	7033 (10.5)	581 (10.7)	
Rectum	9167 (12.7)	8468 (12.6)	699 (12.9)	
Colon	50,677 (69.9)	46,882 (70)	3795 (69.9)	

Data are expressed as *n* (%) unless otherwise specified

*IQR* interquartile range, *CCI* Charlson Comorbidity Index, *SVI* social vulnerability index

### Covariates

Covariates included patient age, sex, Charlson Comorbidity Index (CCI), race/ethnicity (categorized as White, Black, Hispanic, or other [with the ‘other’ category encompassing Asian, American Indian, and Alaska Native]), area of residence (metropolitan vs. non-metropolitan), social vulnerability index (SVI), year of diagnosis, cancer type, and admission type (urgent vs. elective); hospital-level data included teaching status. Data on SVI were obtained from the Centers for Disease Control and the Agency for Toxic Substances and Disease Registry.^15^ SVI is a validated measure, developed by the CDC, that uses 18 community factors and evaluates the susceptibility of communities to certain external stressors.

### Outcomes of Interest

The primary outcomes were HDAH and 30-day readmission. HDAH is a novel population-based quality measure developed by the Medicare Payment Advisory Commission. HDAD is a proxy for quality of life and measures the days an individual spends outside a healthcare facility, usually at home with family or friends.^16^ To calculate the 90-day HDAH, the number of days the patient spent in inpatient care, observation, skilled nursing facilities (SNFs), outpatient emergency departments, inpatient psychiatry, and inpatient rehabilitation were subtracted from the initial 90 days otherwise spent at home post-discharge.^16^ Secondary outcomes included extended length of stay, complications, discharge disposition, and 30-day and 1-year postoperative expenditures. Extended length of stay was defined as an inpatient stay greater than the 75th percentile. Expenditures were adjusted for geographic variation using the wage index and for inflation using the healthcare price index for 2020.

### Statistical Analysis

Descriptive statistics were presented as median values with interquartile range (IQR) for continuous variables and as frequency (%) for categorical variables. Differences in baseline characteristics were assessed using the Kruskal–Wallis test for continuous variables and either the Chi-square test or Fisher’s exact test for categorical variables. Multivariable logistic regression models were utilized to examine the association between disability and outcomes; odds ratios (OR) and 95% confidence intervals (CI) were reported. A multivariable generalized linear model with a gamma distribution and log link was fitted to examine healthcare expenditures among patients with and without disabilities. The models were adjusted for age, sex, ethnicity, region, area, hospital setting, procedure type, CCI, and SVI. Statistical tests were conducted using a two-tailed approach at a significance level of *p* < 0.05. Analyses were performed using SAS 9.4 (SAS Institute, Inc., Cary, NC, USA).

## Results

### Baseline Characteristics

A total of 72,452 Medicare beneficiaries underwent a surgical procedure for a GI cancer (pancreas: *n* = 7614, 10.5%; liver: *n* = 2345, 3.2%; biliary tract: *n* = 2649, 3.7%; colon: *n* = 50,677, 69.9%; rectum: *n* = 9167, 12.7%) during the study period. Median patient age was 75 years (IQR 71–81) with approximately half of the patients being female (*n* = 37,167, 51.3%). Most individuals had a high CCI score (CCI ≥2: *n* = 39,315, 54.3%) and underwent surgery at a teaching hospital (*n* = 44,242, 61.1%). A majority of the cohort self-identified as White (*n* = 64,152, 88.5%), with fewer individuals identifying as Black (*n* = 4217, 5.8%), Hispanic (*n* = 599, 0.4%), or ‘other’ race (*n* = 3484, 4.8%). Most individuals resided in the South (*n* = 28,994, 40.0%) or Midwest (*n* = 17,460, 24.1%), followed by the Northeast (*n* = 13,522, 18.7%) and West (*n* = 12,476, 17.2%) regions of the US. Overall, 5432 patients (7.5%) had a disability. Patients with disabilities were more likely to be older (disability: 77 years [IQR 72–82] vs. no disability: 75 years [IQR 71–80]) and had higher CCI scores (CCI >2; disability: 63.9% vs. no disability: 53.5%) [both *p* < 0.001]. Additionally, Black (disability: 6.9% vs. no disability: 5.7%) and Hispanic individuals (disability: 1.1% vs. no disability: 0.8%) were more likely to have a disability (*p* < 0.001) (Table 1).

### Disability and Surgical Outcomes

**Fig. 1:**
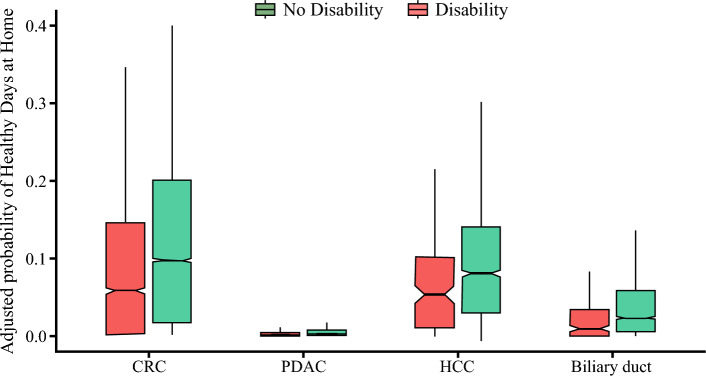
Risk-adjusted probability of spending higher (>80th percentile) healthy days at home, stratified by cancer type. CRC colorectal cancer, PDAC pancreatic ductal adenocarcinoma, HCC hepatocellular carcinoma

Following the index surgery, 12.4% (*n* = 8951) of patients had an extended length of hospital stay, 3.4% (*n* = 2477) experienced complications, and 14.0% (*n* = 10,119) were readmitted within 30 days. Moreover, most patients were discharged home (*n* = 41,944, 57.9%), while a small subset was discharged to an SNF (*n* = 9714, 13.4%). The median 30-day expenditure was $20,899 (IQR 15,652–32,465), and the 1-year expenditure was $35,154 (IQR 20,640–62,701). Of note, individuals with a disability were more likely to be discharged to an SNF (disability: 26.6% vs. no disability: 12.3%) and had fewer HDAH (<20th percentile) [disability: 33.0% vs. no disability: 19.2%] (both *p* < 0.001). Likewise, patients with disability were less likely to spend a high number (80th percentile) of days at home (disability: 15.0% vs. no disability: 7.6%; *p* < 0.001) (Fig. 1). Moreover, patients with a disability were more likely to experience complications (disability: 4.6% vs. no disability: 3.3%), have an extended length of stay (disability: 16.3% vs. no disability: 12.0%), and readmission within 30 days (disability: 20.0% vs. no disability: 13.5%) [all *p* < 0.001]. Median expenditures at 30 days (disability: $24,768 [IQR 17,318–38,497] vs. no disability: $20,631 [IQR 15,534–32,006]) and 1 year (disability: $49,968 [IQR 28,489–85,882] vs. no disability: $34,212 [IQR 20,297–60,890]) were also higher among individuals with a disability (both *p* < 0.001) (Table 2). An additional analysis stratified by the type of disability demonstrated similar results; specifically, individuals with motor or visual impairments experienced higher complications, spent fewer days in HDAH, and had a lower likelihood of being discharged to home (ESM Table 3).

**TABLE 2:** Surgical outcomes in patients with and without disability

Outcomes	Total [*n* = 72,452]	Disability		*p* value
		No [*n* = 67,020, 92.5%]	Yes [*n* = 5432, 7.5%]	
30-day readmission	10,119 (14)	9032 (13.5)	1087 (20)	<0.001
90-day readmission	16,880 (23.3)	15,133 (22.6)	1747 (32.2)	
Discharge disposition				<0.001
HHA	18,079 (25)	16,711 (24.9)	1368 (25.2)	
Home	41,944 (57.9)	39,591 (59.1)	2353 (43.3)	
Other	2715 (3.7)	2448 (3.7)	267 (4.9)	
SNF	9714 (13.4)	8270 (12.3)	1444 (26.6)	
90-day complication	3804 (5.3)	3415 (5.1)	389 (7.2)	<0.001
30-day complication	2477 (3.4)	2227 (3.3)	250 (4.6)	<0.001
Healthy days at home^a﻿^				<0.001
Low	14,660 (20.2)	12,865 (19.2)	1795 (33)	
Moderate	47,331 (65.3)	44,108 (65.8)	3223 (59.3)	
High	10,461 (14.4)	10,047 (15)	414 (7.6)	
Extended length of stay	8951 (12.4)	8063 (12)	888 (16.3)	<0.001
Expenditure postoperatively 1 year, $ (IQR)	35,154 (20,640–62,701)	34,212 (20,297–60,890)	49,968 (28,489–85,882)	<0.001
Expenditure postoperatively 30 days, $ (IQR)	20,899 (15,652–32,465)	20,631 (15,534–32,006)	24,768 (17,318–38,497)	<0.001

^a^Healthy days at home 90 days postoperatively

*HHA* home health assistance, *SNF* skilled nursing facility, *IQR* interquartile range

**Table 3:** Multivariable analysis examining the association between disability and surgical outcomes (reference: no disability)

Outcomes	OR	95% CI
30-day readmission	1.55	1.44–1.66
90-day readmission	1.57	1.48–1.67
Disposition – home	0.56	0.52–0.59
90-day complication rate	1.39	1.24–1.55
30-day complication rate	1.36	1.19–1.56
Healthy days at home^a^	1.88	1.77–1.99
Extended length of stay	1.32	1.22–1.43

^a^Healthy days at home 90 days postoperatively, odds of spending fewer days (low) at home

*OR* odds ratio, *CI* confidence interval

**Fig. 2:**
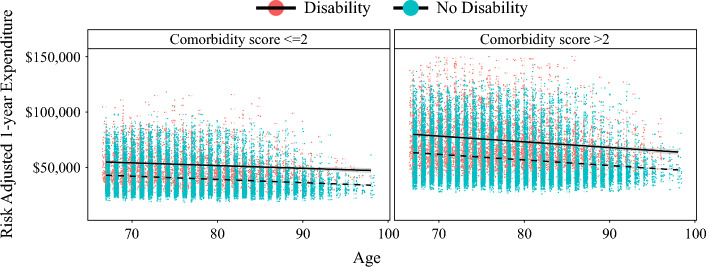
Risk-adjusted 1-year expenditures among patients with and without disability, stratified by Charlson Comorbidity Index score

On multivariable analysis, disability remained independently associated with surgical outcomes and fewer HDAH. Notably, disability was associated with higher odds of experiencing postoperative complications (reference: no disability; OR 1.36, 95% CI 1.19–1.56), an extended length of stay (reference: no disability; OR 1.32, 95% CI 1.22–1.43), 30-day readmission (reference: no disability; OR 1.55, 95% CI 1.44–1.66), and fewer HDAD (reference: no disability; OR 1.88, 95% CI 1.77–1.99) (Table 3). Additionally, individuals with disabilities had 14.22% higher expenditures for the 30-day postoperative period (95% CI 1.25–1.40) and 35.05% higher expenditures for the 1-year postoperative period (95% CI 1.25–1.40) (Fig. 2 and ESM Table 2).

## Discussion

Disability and its impact on health management is a growing concern in the US. With the increase in the aging population, the number of patients with disabilities in the US has also risen.^14^ As such, individuals with disabilities represent one of the largest underserved groups in the US, and there is a growing demand to address the unmet needs of this group. Previous studies have noted that individuals with disabilities are less likely to utilize screening and preventive services and experience poorer outcomes following hospitalization for chronic medical conditions.^18,19^ Nevertheless, the impact of disability on outcomes following GI cancer surgery remains poorly defined. Of note, cancer surgery is highly invasive, and postoperative care requires adherence to intricate treatment plans, which can be challenging for individuals with disabilities, who also often lack capacity of self-care.^20,21^ Therefore, the current study was important as it specifically examined the association between disability and postoperative outcomes using a large nationally representative database. Notably, disability was independently associated with higher odds of experiencing postoperative complications, an extended length of stay, and readmission within 30 days. Additionally, individuals with a disability were less likely to be discharged home and spent less HDAD. The current study highlights the need for targeted interventions to improve the care of patients with disabilities and ensure equitable oncological outcomes.

The poor surgical outcomes noted in the current study may be partially attributed to the barriers that individuals with disabilities face when navigating the healthcare system. Many patients with a disability face unique challenges, in understanding their health status, identifying the need to take action, and seeking timely intervention.^19,20^ Delayed presentation of cancer could lead to worse outcomes.^24^ Moreover, disabilities that impact mobility may cause hindrances in reaching hospitals and utilizing institutional resources such as weight scales, height-adjustable examination tables, accessible parking, and ramp access.^25,26^ During interactions with providers, communication barriers can hinder patients’ complete understanding, particularly for those with diminished cognitive abilities or those requiring interpreters, such as individuals with visual or hearing impairments.^27,28^ Compliance and effective postoperative care can also have its own challenges. Motor impairment causes decreased mobility, which is a negative indicator for surgical outcomes, including higher complication, readmission, and mortality.^29^ Visual impairment could impede proper wound care for a self-reliant patient, with limited capacity to assess the wound.^30^ For instance, Venishetty et al. noted that individuals with legal blindness have poor surgical outcomes, including increased complications and extended length of stay.^31^ The healthcare system requires careful attention and planning to better accommodate individuals with disabilities.

Previous reports have studied surgical outcomes in terms of single events, such as complications, hospital readmission, or mortality.^32^ Mortality is an objective, patient-centered outcome measure; however, it is relatively blunt and does not capture other, less severe harms that are also important to patients and their families. Moreover, focusing on complications and hospital readmissions carries the risk of incentivizing a narrow focus at the expense of other, potentially more important outcomes, such as mortality. As such, the current study utilized a novel metric, HDAD, which represents the number of days spent alive and outside of facility-based healthcare settings. This metric serves as a proxy for quality of life following surgery. Of note, the current dataset demonstrated that following GI cancer surgery, patients with disabilities spend fewer HDAD and spend most of their time in a hospital or an SNF. The increased rates of complications, hospital readmissions, and discharges to SNFs impact the patient’s quality of life and also add financial burden to the healthcare system. In 2015, disability-associated healthcare expenditure for people residing in the US totaled $868 billion, with an average expenditure of $17,431 per person with a disability.^33^ This excess burden on both the patient and the healthcare system needs to be addressed by implementing systems that provide more targeted postoperative care, rehabilitation, and management for patients with varying accessibility requirements.^34^

The healthcare system needs to adopt innovative strategies to better address and prioritize the needs of high-risk, vulnerable communities, such as individuals with disabilities. Healthcare providers should have more awareness when understanding disability and the consequent inequity and social implications as a whole.^35^ Moreover, healthcare workers should receive detailed training on assessing and addressing the needs of individuals with disabilities.^36,37^ To improve accessibility, improving the delivery of information about consent, disease, and management is key.^38^ Moreover, it is essential to assist visually- and hearing-impaired personnel in navigating unfamiliar surroundings and using work equipment, such as weighing scales and examination tables, in a manner that is accessible and universally appropriate for various disabilities.^23^ Programs such as the National Association of County and City Health Officials (NACCHO) are using ‘disability specialists’ to work with local health departments to analyze and ensure disability inclusion and increase collaboration between local health departments and disability organizations. There is a need for such organizations to expand and improve accessibility across more health centers and hospitals.^39^

The findings of the current study should be interpreted in light of several limitations. Similar to other studies using large national registries, this analysis may have been affected by coding errors, inaccurate data entry, and incomplete information. As the Medicare database was queried for data, the retrospective nature, and reliance on administrative database, there was potential for residual confounding bias. The study included Medicare beneficiaries aged 65 years and older; hence, the findings may be limited in its generalizability to younger populations or people with different insurance coverages. The current study included various GI cancers, however most of the cohort had colorectal cancer. This was likely due to the higher incidence of colorectal cancer and may potentially limit the generalizability to other cancer types. Moreover, data could not be assessed on an individual, granular basis (e.g., the degree of disability, type of resection, and severity of complications).

## Conclusion

Disability was associated with postoperative complications, an extended length of stay, and readmission within 30 days. Moreover, individuals with a disability spent fewer HDAD. These data highlight the need to improve the care of patients with disabilities to ensure equitable oncologic surgical care.

## Supplementary Information

Below is the link to the electronic supplementary material.

Supplementary file1 (DOCX 18 KB)

## Data Availability

The data for this study were obtained from the Medicare database. There are restrictions to the availability of this data, which is used under license for this 12 study. Data can be accessed with permission from the Centers for Medicare and Medicaid Services.
