# Supplementary material for: Impact of Disability on Postoperative Outcomes After Gastrointestinal Cancer Surgery
Source: Ann Surg Oncol. 2025 Jan 16;32(5):3352–9. doi: 10.1245/s10434-025-16904-x (PMC11976814; doi:10.1245/s10434-025-16904-x)
Supplement: Supplementary file 1 — Supplementary file1 (DOCX 18 KB) [file 10434_2025_16904_MOESM1_ESM.docx]

**Supplementary Table 1:** ICD-10 codes used to identify disability conditions.

| **ICD-10 Codes** | **Disabilities** |
| --- | --- |
| **Hearing Impairment** | |
| F446 | psychogenic deafness |
| H93.2 | Other abnormal auditory perceptions. |
| H90.0 | Conductive hearing loss, bilateral |
| H90.1 | Conductive hearing loss, unilateral with unrestricted hearing on the contralateral side |
| H90.4 | Sensorineural hearing loss, unilateral with unrestricted hearing on the contralateral side. |
| H90.2 | Conductive hearing loss, unspecified |
| H90.3 | Sensorineural hearing loss, bilateral. |
| H90.5 | Sensorineural hearing loss, unspecified. |
| H90.6 | Mixed conductive and sensorineural hearing loss, bilateral. |
| H90.7 | Mixed conductive and sensorineural hearing loss, unilateral with unrestricted hearing on the contralateral side. |
| H90.8 | Mixed conductive and sensorineural hearing loss, unspecified. |
| H91.9 | Unspecified hearing loss |
| Q16 | Congenital malformations of ear causing impairment of hearing. |
| **Motor Impairment** | |
| Z736 | Limitation of activities due to disability |
| Z740 | Reduced mobility |
| Z7401 | Bed confinement status |
| Z7409 | Other reduced mobility |
| Z741 | Need for assistance with personal care. |
| Z742 | Need for assistance at home and no other household member able to render care. |
| Z743 | Need for continuous supervision |
| Z748 | Other problems related to care provider dependency |
| Z749 | Problem related to care provider dependency, unspecified |
| Z993 | Dependence on wheelchair |
| **Visual Impairment** | |
| H540 | Blindness, both eyes |
| H541 | Blindness, right eye, normal vision left eye. |
| H542 | Low vision, both eyes |
| H543 | Unqualified visual loss, both eyes. |
| H544 | Blindness, one eye. |
| H545 | Severe visual impairment, monocular |
| H546 | Unqualified visual loss, one eye |
| H547 | Unspecified visual loss |
| H548 | Legal blindness |
| H530 | Amblyopia ex anopsia |
| H531 | Subjective visual disturbances |
| H535 | Color vision deficiencies |
| H536 | Night blindness |
| H538 | Other visual disturbances |
| **Intellectual Disability** | |
| F70 | Mild intellectual disability |
| F71 | Moderate intellectual disability |
| F72 | Severe intellectual disability |
| F73 | Profound intellectual disabilities |
| F78 | Other intellectual disabilities |
| F79 | Unspecified intellectual disabilities |
| Q90 | Down Syndrome |

**Supplementary Table 2:** Percentage difference in expenditures

| **Outcomes** | **Percent difference** | **95% CI** |
| --- | --- | --- |
| Expenditure postop 30 days | 14.22 | 12.76-15.70 |
| Expenditure postop 1 year | 35.05 | 32.67-37.48 |

**Supplementary Table 3.** Surgical outcomes in patients with motor and visual impairment.

| **Outcomes** | **Total**  **(n=72,452)** | **Disability** | | **p value** |
| --- | --- | --- | --- | --- |
|  |  | **No** | **Yes** |  |
| Discharge to Home  Visual  Motor | 41,944 (57.9) | 41,157 (58.1) 40,904 (58.8) | 787 (48.9) 1,040 (36.7) | <0.001 <0.001 |
| 90-day complication  Visual  Motor | 3,804 (5.3) | 3,715 (5.2) 3,541 (5.1) | 89 (5.5) 263 (9.3) | 0.609 <0.001 |
| Healthy days at home*  Low  Visual  Motor  Moderate  Visual  Motor  High  Visual  Motor | 14,660 (20.2)  47,331 (65.3)  10,461 (14.4) | 14,233 (20.1)  13,495 (19.4)  46,302 (65.4)  45,833 (65.8)  10,308 (14.6) 10,289 (14.8) | 427 (26.5) 1,165 (41.0)  1,029 (64.0)  1,498 (52.8)  153 (9.5) 172 (6.0) | <0.001  <0.001   <0.001 |

^*Healthy days at home 90 days post-operatively^
